# Serum phosphate levels and the development of sepsis associated acute kidney injury: evidence from two independent databases

**DOI:** 10.3389/fmed.2024.1367064

**Published:** 2024-03-22

**Authors:** Yipeng Fang, Yuan Zhang, Xin Zhang

**Affiliations:** ^1^Laboratory of Molecular Cardiology, The First Affiliated Hospital of Shantou University Medical College, Shantou, China; ^2^Laboratory of Medical Molecular Imaging, The First Affiliated Hospital of Shantou University Medical College, Shantou, China; ^3^Shantou University Medical College, Shantou, China

**Keywords:** phosphorus, hyperphosphatemia, hypophosphatemia, acute renal failure, novel biomarker, prediction, risk factor

## Abstract

**Objective:**

We aimed to investigate the association between serum phosphate levels and the risk for developing sepsis associated acute kidney injury (SAKI).

**Methods:**

Septic patients from the Medical Information Mart for Intensive Care IV (MIMIC IV) and the eICU Collaborative Research Database (eICU-CRD) were enrolled. Restricted cubic spline (RCS) was used to visualize the relationship between phosphate levels and the risk of SAKI. Patients were divided into four categories based on their serum phosphate levels. Logistic regression analysis, receiver operating characteristic (ROC) curve and subgroup analysis were performed to evaluate the predictive value of serum phosphate for SAKI.

**Results:**

A total of 9,244 and 2,124 patients from the MIMIC IV and eICU-CRD database were included in the final analysis. RCS curve revealed a non-linear correlation between phosphate levels and the risk of SAKI (*p* for non-linearity <0.05). Each 1 mg/dL increase in phosphate levels was associated with a 1.51 to 1.64-fold increased risk of SAKI (OR 2.51–2.64, *p* < 0.001) in the MIMIC IV cohort and a 0.29 to 0.38-fold increased risk (OR 1.29–1.38, *p* < 0.001) in the eICU-CRD cohort. Compared to the normal-low category, hyperphosphatemia and normal-high category were independently associated with an increased risk of SAKI, while hypophosphatemia was independently associated with a decreased risk in the MIMIC IV cohort. A similar trend was observed in the eICU-CRD cohort, but statistical significance disappeared in the hypophosphatemia category and the adjusted model of normal high category. These finding was consistent in subgroup analysis.

**Conclusion:**

Elevated serum phosphate, even within the normal range, is an independent risk factor for developing SAKI in septic patients. Abnormal change in serum phosphate levels may be a novel biomarker for early prediction of SAKI occurrence.

## 1 Introduction

Sepsis is a life-threatening condition characterized by organ dysfunction of caused by an imbalanced immune response to infection (1). It leads to abnormalities in circulation, cellular metabolism, and significant increase of mortality risk for certain patients (1). Currently, sepsis remains a major global health concern, with more than 19 million cases diagnosed annually, particularly in underdeveloped regions (2, 3). Sepsis-associated acute kidney injury (SAKI) is a common complication with an incidence ranging from 23 to 51%, which contributes to the increased mortality in septic patients. Previous studies showed that SAKI accounted for over 50% of all cases of acute kidney injury (AKI) (4, 5). Furthermore, patients with SAKI face a substantially higher risk of death compared to those with non-sepsis related AKI (6). Early prediction and identification of SAKI are crucial for effective treatment and better outcomes. Consequently, extensive attention has been devoted to discover biomarkers that can facilitate the early recognition of SAKI.

Phosphate is a mineral that is widely distributed in nature and is the second most abundant mineral in the human body, accounting for approximately 1% of total body weight (7). It serves as a vital structural component of bones, teeth and DNA/RNA, making lipid membranes and circulating lipoproteins bipolar (8). Additionally, phosphate plays key roles in different biological processes such as energy generation and storage (formation of a phosphate bond in ATP), pH buffering in blood, regulation of gene expression, enzyme activation, molecule modification, and subsequently affecting a variety of organ functions from renal excretion to immune response (8).

Several studies suggested the association between abnormal changes of serum phosphate levels and the prognosis of patients with sepsis (9–11). Li et al. (9) found a nearly positive linear relationship between serum phosphate levels and the risk of death in patients with sepsis. Similarly, Xu et al. (10) demonstrated that hypophosphatemia might be an independent protective factor, while hyperphosphatemia might be an independent risk factor for 28 days mortality in septic patients. They also suggested that serum phosphate levels obtained on the second day of ICU admission have a higher predictive value for 28 days mortality compared to those obtained on the first day. Furthermore, Shor et al. (11) discovered that severe hypophosphatemia (serum inorganic phosphate <1 mg/dL) increases the risk of mortality by almost eightfold in septic patients compared to those without severe hypophosphatemia.

These findings highlight the importance of serum phosphate levels as potential biomarkers for assessing prognosis in septic patients. However, as a potential prognosis biomarker of sepsis, the association between serum phosphate and the risk of developing SAKI in patients with sepsis has not received enough attention. In this study, we aimed to explore the potential association between serum phosphate levels and the incidence of SAKI using data from two publicly available database: the Medical Information Marketplace in Intensive Care IV (MIMIC-IV) database and the eICU Collaborative Research Database (eICU-CRD). Our study provides new important clues of serum phosphate in early diagnosis of SAKI.

## 2 Materials and methods

### 2.1 Data sources

This multiple-center observational study utilized data from two independent large public clinical databases, namely the MIMIC-IV (12) and the eICU-CRD (13). The MIMIC-IV database contains hospital records of patients admitted to the Beth Israel Deaconess Medical Center (BIDMC) between 2008 and 2019. The eICU-CRD database includes over 200,000 intensive care units (ICUs) admission records from various locations across the United States between 2014 and 2015. Access to these databases was granted to YF (Record ID: 43025968) after completing the National Institutes of Health (NIH) training course and passing the Protecting Human Research Participants test. The present study was conducted in accordance with the guidelines outlined in the Helsinki Declaration. Ethics approval for the MIMIC database was granted by the Massachusetts Institute of Technology and the Institutional Review Board of BIDMC. Informed consent was waived due to anonymized nature of the data. In addition, no additional institutional review board approval was required for the study of the eICU-CRD database (information available at: https://eicu-crd.mit.edu/about/acknowledgments/). This manuscript was prepared following the Strengthening the Reporting of Observational Studies in Epidemiology (STROBE) statement (statement available at: https://www.strobe-statement.org/).

### 2.2 Study population

Adult patients with sepsis during their ICU stay were selected from the MIMIC IV and eICU-CRD, respectively. The screening criteria for septic patients were based on the Sepsis 3.0 definition (1) Patients were excluded if they met any of the following criteria: (1) age <18 years; (2) multiple hospital admissions, except for their initial ICU admission; (3) development of AKI prior to sepsis diagnosis (4) missing phosphate results at the time of sepsis diagnosis; (5) with hemodialysis use before SAKI development (6) presence of chronic kidney disease or parathyroid dysfunction; (7) ICU stay less than 48 h.

### 2.3 Exposure and endpoints

Serum phosphate results obtained within 24 h prior to the diagnosis of sepsis were utilized as the exposure variable. Patients was further divided into several subgroups based on the normal reference ranges and the optimal cut-off value suggested by the restricted cubic spline (RCS) curve. The normal reference ranges were determined as 2.7 mg/dL–4.5 mg/dL according to the reference values from the MIMIC-IV database.

The primary outcome was the development of AKI within 48 h of sepsis diagnosis. AKI was determined using the creatinine criteria of KDIGO (14). Secondary outcomes included the use of continuous renal replacement therapy (CRRT) after the diagnosis of SAKI, the mortality and length of stay (LOS). Notedly, to minimize the influence of AKI on blood phosphate levels, the evidences of exposure and outcome variables were obtained before and after the diagnosis of sepsis, respectively. Additionally, patients who developed AKI prior to the occurrence of sepsis were excluded from the analysis.

### 2.4 Data extraction

The following structured data were extracted from the MIMIC-IV and eICU-CRD databases by PostgreSQL (version 9.6) and PgAdmin4 software: (1) demographic variables included age, sex, ethnicity, weight; (2) comorbidities included hypertension, heart failure, coronary heart disease, diabetes mellitus, chronic kidney disease, chronic pulmonary disease, liver disease and malignant cancer; (3) SOFA score at the time of sepsis diagnosis; (4) laboratory parameters of blood samples included the mean value of white blood cells (WBC), hemoglobin, platelets, sodium, potassium obtained within 48 h of sepsis diagnosis; the maximum value of serum creatinine and blood urea nitrogen (BUN) within 48 h of sepsis diagnosis; (5) the minimum value of mean blood pressure (MBP) within the 48 h of sepsis diagnosis; (6) treatment received: mechanical ventilation and vasoactive agent use from ICU admission until SAKI occurred. Vasoactive agent was identified as the use of norepinephrine, epinephrine, vasopressin, dopamine and dobutamine. Although lactate level is an important prognostic biomarker in patients with sepsis, it was not included in the final analysis due to a high proportion of missing values (over 45%).

### 2.5 Data clean

Scatter plots for bivariate correlations were performed to identify outliers. Any outliers detected were treated as missing values. Continuous variable data with a missing value percentage exceeding 10% were excluded from the analysis. For continuous variable data with missing values below 10%, these values were replaced with the mean or median according to their distribution, as appropriate. The proportion of missing values for all remaining data used in the final analysis is less than 10%.

### 2.6 Statistical analysis

For continuous variable data with normal distribution, data was displayed as mean ± standard deviation (SD) and further analyzed using student’s *t*-test in two groups comparison. For continuous variable data with non-normal distribution, data was displayed as median (interquartile range, IQR) and further analyzed using Mann–Whitney *U*-test in two groups comparison. One-way ANOVA (for normally distributed values) or Kruskal–Wallis *H* test followed by Bonferroni post hoc tests (Correction = 0.05/6) was employed for multiple comparisons of continuous variables with normally and non-normally distributed values, respectively. Data for categorical variables were reported as numbers (percentages). The chi-square test was conducted to analyze categorical variable data. Restricted cubic spline (RCS) analysis was utilized to determine and visualize the correlation between serum phosphate levels and the risk of developing SAKI. Logistic regression analysis was performed to investigate the predictive value of serum phosphate for the risk for developing SAKI in crude and adjusted models. Multicollinearity was assessed using the variance inflation factor (VIF), and parameters with a VIF ≥10 were removed from the model due to multicollinearity issues. The clinical predictive value was further evaluated using receiver operating characteristic (ROC) curve analysis, with the area under the ROC curve (AUC) representing the clinical value. Subgroup analysis was conducted to explore potential interactions and assess the robustness of the findings. A significance level of <0.05 (two-tailed) was considered statistically significant. All statistical analyses were performed using Stata 15 and R software version 4.1.3.

## 3 Results

### 3.1 Baseline information and clinical results from the MIMIC IV and eICU-CRD databases

**Figure 1:**
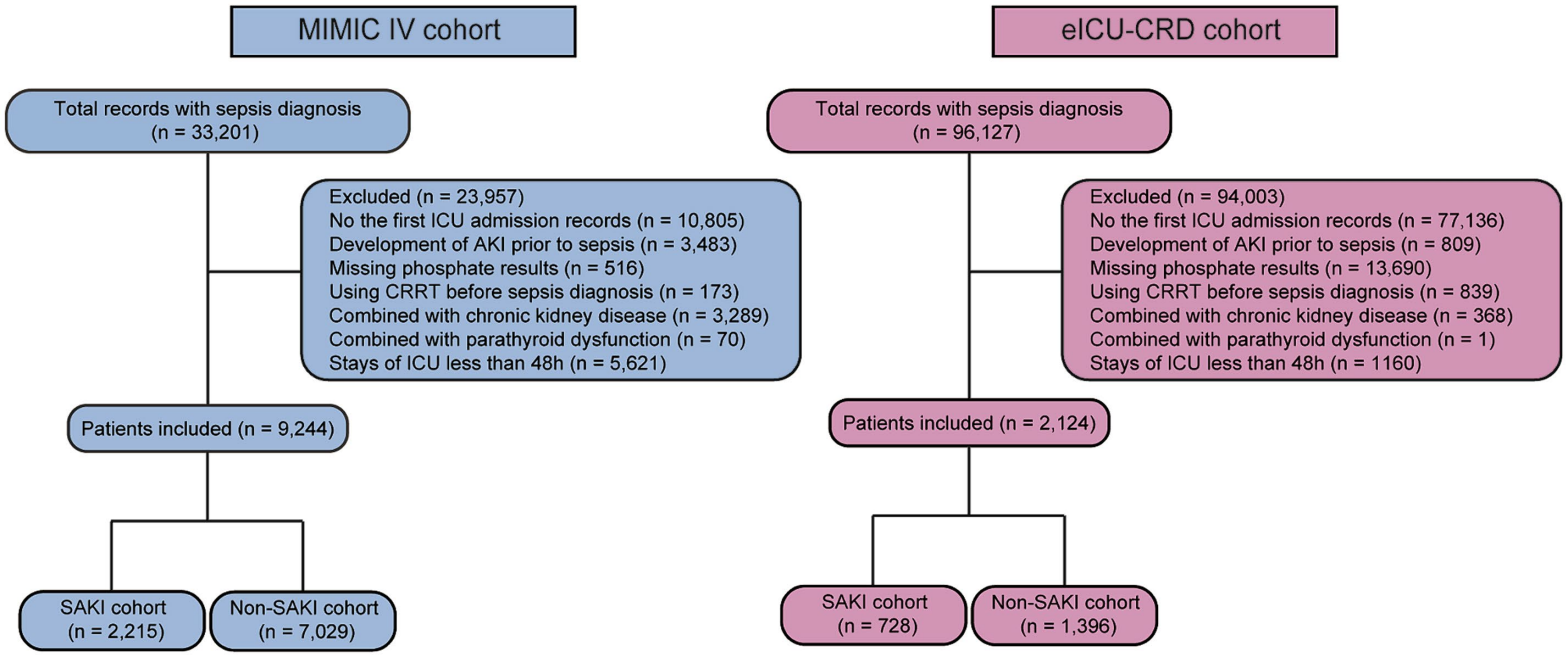
The flow chart of patient selection in the MIMIC IV and eICU-CRD databases. CRRT, continuous renal replacement therapy; ICU, intensive care unit; SAKI, sepsis associated acute kidney injury.

A total of 11,368 eligible patients were included in this study, including 9,244 in the MIMIC IV cohort and 2,124 in the eICU-CRD cohort (see Figure 1). In the MIMIC IV cohort, 2,215 patients (24.0%) developed SAKI within 48 h of sepsis diagnosis (see Table 1). Patients with SAKI were found to be older and heavier compared to those without SAKI (all *p* ≤ 0.001). There was a higher proportion of males in the SAKI subgroup (*p* < 0.001). The proportions of coronary heart disease, heart failure, hypertension, diabetes mellitus, and liver disease were significantly higher in patients with SAKI (all *p* < 0.001). SAKI patients also exhibited higher levels of WBC, potassium, creatinine, and BUN, but lower levels of hemoglobin, platelets and sodium (all *p* < 0.05). The MBP was lower in the SAKI subgroup compared to the non-SAKI subgroup (*p* < 0.001). More patients in the SAKI subgroup received more mechanical ventilation and vasoactive drug treatment (all *p* < 0.001). SAKI patients also had the higher SOFA scores (3[2,5] vs. 3[2,4], *p* < 0.001). Serum phosphate levels were higher in patients with SAKI (3.70 ± 0.91 vs. 3.22 ± 0.58, *p* < 0.001).

**Table 1:** Baseline information and clinical outcomes in patients with and without SAKI.

Variable	All patients		MIMIC IV database			eICU database		
	MIMIC IV	eICU	Non-SAKI	SAKI	*p*-value	Non-SAKI	SAKI	*p*-value
Number	9,244	2,124	7,029	2,215		1,396	728	
Age (years)	64.31 ± 15.58	64.26 ± 16.29	63.65 ± 17.28	66.37 ± 15.58	<0.001	64.57 ± 16.51	63.67 ± 15.86	0.231
Male (%)	5,154 (55.76)	1,087 (51.18)	3,832 (54.52)	1,322 (59.68)	<0.001	712 (51.00)	375 (51.51)	0.824
Ethnicity, white (%)	5,847 (63.25)	1,690 (79.57)	4,450 (63.31)	1,397 (63.07)	0.839	1,113 (79.73)	577 (79.26)	0.799
Weight (kg)	82.17 ± 25.18	81.81 (27.49)	81.49 ± 25.31	84.33 ± 24.62	<0.001	80.31 (26.88)	84.69 (28.44)	<0.001
Coronary heart disease (%)	1,498 (16.21)	232 (10.92)	995 (14.16)	503 (22.71)	<0.001	145 (10.39)	87 (11.95)	0.273
Heart failure (%)	2,112 (22.85)	269 (12.66)	1,466 (20.86)	646 (29.16)	<0.001	180 (12.89)	89 (12.23)	0.660
Hypertension (%)	4,915 (53.17)	294 (13.84)	3,643 (51.83)	1,272 (57.43)	<0.001	198 (14.18)	96 (13.19)	0.528
Diabetes mellitus (%)	2,285 (24.72)	367 (17.28)	1,633 (23.23)	652 (29.44)	<0.001	234 (16.76)	133 (18.27)	0.383
Chronic pulmonary disease (%)	2,391 (25.87)	391 (18.41)	1800 (25.61)	591 (26.68)	0.314	286 (20.49)	105 (14.42)	0.001
Liver disease (%)	1,316 (14.24)	250 (11.77)	869 (12.36)	447 (20.18)	<0.001	140 (10.03)	110 (15.11)	0.001
Malignant cancer (%)	1,132 (12.25)	84 (3.95)	859 (12.22)	273 (12.33)	0.896	53 (3.80)	31 (4.26)	0.604
White blood cell (k/μL)	11.6 (8.4, 15.8)	12.6 (8.5, 18.4)	11.5 (8.4, 15.4)	12.5 (8.7, 17.1)	<0.001	12.4 (8.5, 17.4)	13.3 (8.6, 20.0)	0.003
Hemoglobin (g/dL)	10.62 ± 2.02	10.10 ± 1.91	10.64 ± 1.99	10.54 ± 2.13	0.048	10.11 ± 1.88	10.08 ± 1.96	0.784
Platelets (k/μL)	176 (126, 242)	178 (118, 245)	181 (130, 247)	162 (116, 225)	<0.001	183 (125, 253)	169 (106, 229)	<0.001
Sodium (mmol/L)	138.74 ± 5.49	136.12 ± 18.88	138.99 ± 5.37	137.96 ± 5.76	<0.001	138.12 ± 12.38	132.29 ± 26.92	<0.001
Potassium (mmol/L)	4.05 ± 0.64	3.99 ± 0.67	4.00 ± 0.60	4.21 ± 0.73	<0.001	3.93 ± 0.61	4.11 ± 0.74	<0.001
Creatinine (mg/dL)	0.9 (0.7, 1.3)	1.1 (0.7, 2.0)	0.8 (0.7, 1.1)	1.4 (1.1, 2.1)	<0.001	0.9 (0.7, 1.4)	1.8 (1.2, 3.1)	<0.001
Blood urea nitrogen (mmol/L)	20 (14, 30)	26 (16, 41)	18 (13, 26)	28 (20, 40)	<0.001	21 (14, 33)	37 (23, 55)	<0.001
Mean blood pressure (mmHg)	55.81 ± 12.66	57.12 ± 12.28	56.83 ± 12.71	52.58 ± 11.94	<0.001	58.33 ± 12.53	54.80 ± 11.43	<0.001
Mechanical ventilation (%)	5,837 (63.14)	928 (43.69)	4,162 (59.21)	1,675 (75.62)	<0.001	598 (42.84)	330 (45.33)	0.272
Vasoactive drug (%)	3,166 (34.25)	744 (35.03)	2,103 (29.92)	1,063 (47.99)	<0.001	420 (30.09)	324 (44.51)	<0.001
SOFA score	3 (2, 4)	4 (2, 7)	3 (2, 4)	3 (2, 5)	<0.001	3 (2, 6)	6 (3, 8)	<0.001
Phosphate (mg/dL)	3.34 ± 0.70	3.58 ± 1.54	3.22 ± 0.58	3.70 ± 0.91	<0.001	3.31 ± 1.32	4.08 ± 1.78	<0.001

Continuous variables are displayed as mean (standard deviation) or median (first quartile–third quartile); categorical variables are displayed as count (percentage); SOFA, Sequential Organ Failure Assessment.

In the eICU-CRD cohort, the incidence rate of SAKI was 34.3% (728/2124) (shown in Table 1). Patients in the SAKI subgroup had higher body weight (*p* < 0.01). The proportions of chronic pulmonary diseases and liver diseases were lower in the SAKI subgroup (all *p* = 0.001), while the SOFA score was higher (6[3,8] vs. 3[2,6], *p* < 0.001). Similarly, levels of serum WBC, potassium, creatinine, and BUN were higher in patients with SAKI, while platelets and sodium were decreased (all *p* < 0.05). SAKI patients had lower MBP, and received more vasoactive drug treatment (all *p* < 0.001). There were no significant differences between the two groups in terms of age and sex (*p* > 0.05). Patients in the SAKI subgroup had higher serum phosphate levels (4.12 ± 1.72 vs. 3.28 ± 1.25, *p* < 0.001).

### 3.2 Visualization of the correlation between phosphate and the risk of developing SAKI

**Figure 2:**
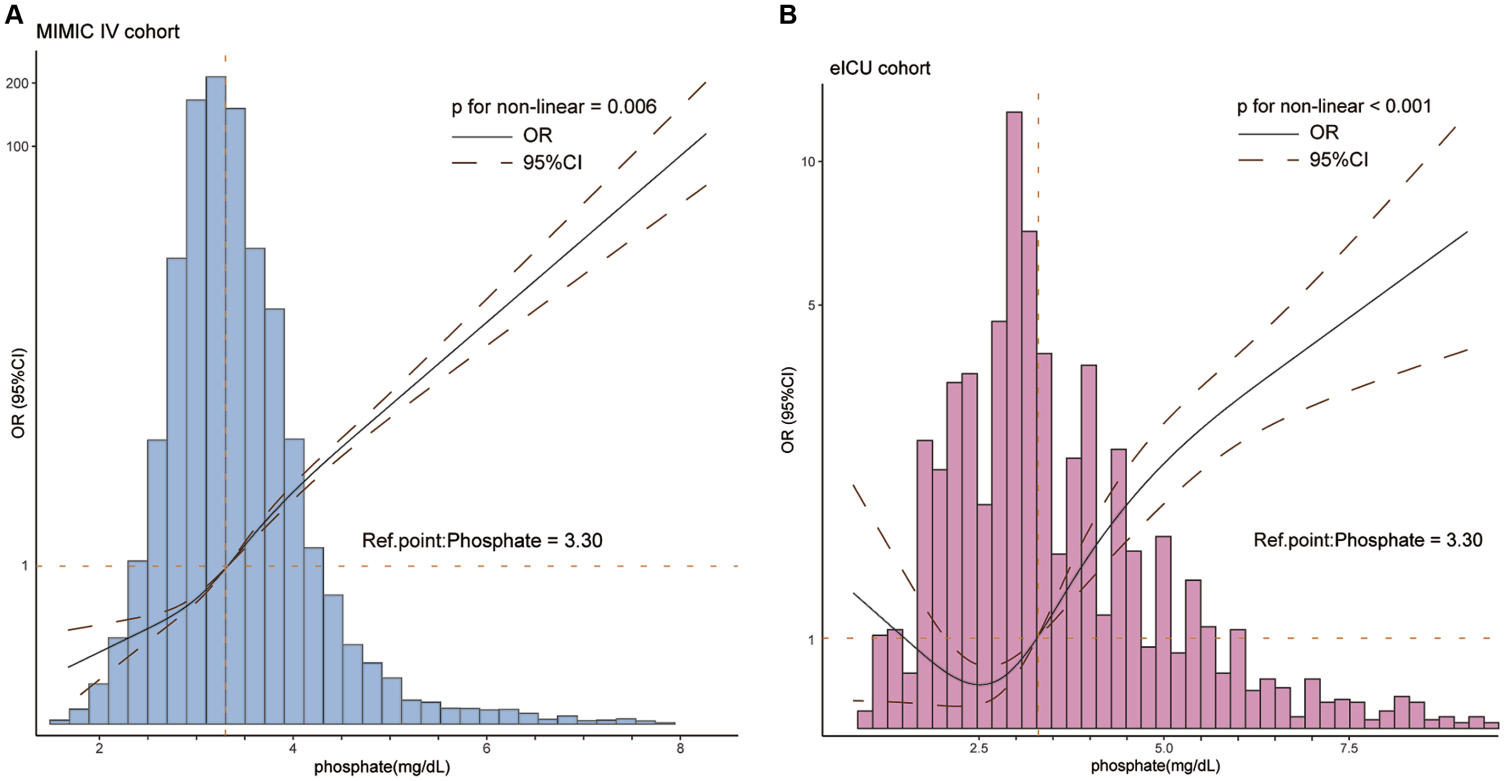
The restricted cubic spline described the non-linear relationship between serum phosphate and the risk of developing SAKI in patients with sepsis in the MIMIC IV **(A)** and eICU-CRD **(B)** cohorts. A positively non-linear correlation between phosphate and the risk of developing SAKI (*p* for non-linearity <0.05) were found in both cohorts. The optimal cut-off value was 3.3 mg/dL. CI, confidence interval; OR, odds ratio. OR is shown as a solid line, CI as a dotted line.

We utilized the RCS method to illustrate the correlation between serum phosphate and the risk of developing SAKI. Figure 2 displays the findings, indicating a positive non-linear relationship between serum phosphate levels and the risk of SAKI in the MIMIC IV cohort (Figure 2A), and a J-shaped relationship was found in the eICU-CRD cohort (Figure 2B), with *p* for nonlinear of 0.06 and <0.001, respectively. The RCS curves suggest that a cut-off value of 3.3 mg/dL is optimal for serum phosphate. Serum phosphate levels above 3.3 mg/dL were considered as an independent risk factor for developing SAKI (OR >1 and lower 95% CI >1). Conversely, serum phosphate levels below 3.3 mg/dL were significantly associated with a reduced risk of SAKI development in both the MIMIC IV and eICU-CRD cohorts (OR <1 and upper 95% CI <1). However, the significant association between serum phosphate and SAKI risk was lost when phosphate levels reached extremely low values in the eICU-CRD cohort (as shown in Figure 2B).

### 3.3 Clinical outcomes of patients in different phosphate categories

It appears that serum phosphate within the normal reference range (2.7 mg/dL–4.5 mg/dL) may have varying predictive values of SAKI development depending on the cut-off value (3.3 mg/dL) obtained from RCS curve. To further investigate the significance of phosphate levels, patients were categorized into four groups based on their phosphate values: hypophosphatemia category (≤2.7 mg/dL), low-normal category (2.7 mg/dL–3.3 mg/dL), high-normal category (3.4 mg/dL–4.5 mg/dL) and hyperphosphatemia category (>4.5 mg/dL).

In both MIMIC IV and eICU-CRD cohorts, we observed a consistent trend of the SAKI incidence across the different categories. Patients in the hyperphosphatemia category had the highest incidence of SAKI, followed by the high-normal, low-normal and hypophosphatemia categories (as shown in Table 2). In the MIMIC IV cohort, significantly differences were found in the comparisons between each category (all adjusted *p* < 0.05). In the eICU-CRD cohort, the incidence of SAKI in patients in the hyperphosphatemia category (54.11%) was significantly higher than that in the other three groups (all adjusted *p* < 0.05). However, there was no statistical difference between the hypophosphatemia and low-normal categories, or between the low-normal and high-normal categories (all adjusted *p* > 0.05).

**Table 2:** Clinical outcomes of patients in different phosphate categories.

Outcomes	Hypophosphatemia (≤2.7 mg/dL)	Normal-low (2.8–3.3 mg/dL)	Normal-high (3.4–4.5 mg/dL)	Hyperphosphatemia (>4.5 mg/dL)	*p*-value
**MIMIC IV database**					
Number	1,293	3,615	3,917	288	
SAKI (%)	167 (12.92)^a^	635 (17.57)^b^	1,125 (28.72)^c^	288 (68.74)^d^	<0.001
Hospital mortality (%)	175 (13.53)^a^	344 (9.52)^b^	426 (10.88)^a,b^	182 (43.44)^c^	<0.001
ICU mortality (%)	130 (10.05)^a^	270 (7.47)^b^	309 (7.89)^a,b^	165 (39.38)^c^	<0.001
Hospital LOS (day)	7.8 (5.5, 11.7)^a^	9.9 (6.6, 15.9)^b^	11.7 (7.1, 19.8)^c^	10.1 (5.3, 17.8)^b^	<0.001
ICU LOS (day)	3.8 (2.7, 6.0)^a^	4.4 (2.9, 7.9)^b^	5.0 (3.1, 10.0)^c^	5.6 (3.6, 10.1)^c^	<0.001
**eICU database**					
Number	617	503	542	462	
SAKI (%)	159 (25.77)^a^	137 (27.24)^a,b^	182 (33.58)^b^	250 (54.11)^c^	<0.001
Hospital mortality (%)	118 (19.12)^a^	89 (17.69)^a^	108 (19.93)^a^	131 (28.35)^b^	<0.001
ICU mortality (%)	83 (13.45)^a^	62 (12.33)^a^	83 (15.31)^a^	99 (21.43)^b^	<0.001
Hospital LOS (day)	10.2 (6.7, 17.5)^a^	10.8 (7.0, 17.9)^a^	10.2 (6.5, 17.2)^a^	10.0 (6.1, 16.8)^a^	0.309
ICU LOS (day)	4.9 (3.0, 8.4)^a^	5.0 (3.0, 9.1)^a^	5.1 (3.1, 8.8)^a^	5.1 (3.1, 9.1)^a^	0.919

Continuous variables are displayed as mean (standard deviation) or median (first quartile–third quartile); categorical variables are displayed as count (percentage). For multiple comparisons among each category, *p*-value was adjusted by Bonferroni method (Correction = 0.05/6), and the statistically significant differences were indicated with superscript letters (Bonferroni-adjusted *p* < 0.05). CRRT, continuous renal replacement therapy; ICU, intensive care unit; LOS, length of stays; SAKI, sepsis-associated acute kidney injury.

Regarding hospital mortality and ICU mortality rates, patients in the hyperphosphatemia category had significantly higher mortality rates compared to those in the other three categories in both the MIMIC IV and eICU-CRD cohorts (all adjusted *p* < 0.05). In the MIMIC cohort, the mortality rates were significantly increased in the hypophosphatemia category, compared to the low-normal category (adjusted *p* < 0.05), but no significant difference was found between the low-normal and the high-normal categories (adjusted *p* > 0.05). In the eICU-CRD cohort, there were no statistically significant differences in mortality rates among the hypophosphatemia, low-normal and the high-normal categories (adjusted *p* > 0.05). We also observed significant differences in hospital and ICU LOS between multiple groups in the MIMIC IV cohort (all *p* < 0.001), but not in the eICU-CRD cohort (*p* = 0.309 and 0.919).

### 3.4 Investigate the predictive value of phosphate for SAKI development using logistic regression analysis

To further investigate the predictive value of phosphate for SAKI development, logistic regression analysis was conducted (as shown in Table 3). Serum phosphate levels were considered to be an independent risk factor for SAKI development, with each 1 mg/dL increase in phosphate levels raising the risk of SAKI development by 1.51 to 1.64-fold (OR 2.51–2.64 in different models, all *p* < 0.001) in the MIMIC IV and by 0.29 to 0.38-fold (OR 1.29–1.38, all *p* < 0.001) in the eICU-CRD cohort.

**Table 3:** Logistic regression analysis for the predictive value of serum phosphate on SAKI.

Phosphate (mg/dL)	MIMIC IV database		eICU database	
	OR (95% CI)	*p*-value	OR (95% CI)	*p*-value
Unadjusted	2.64 (2.44–2.85)	<0.001	1.38 (1.30–1.47)	<0.001
Model 1	2.72 (2.51–2.95)	<0.001	1.38 (1.30–1.47)	<0.001
Model 2	2.55 (2.34–2.78)	<0.001	1.29 (1.21–1.38)	<0.001
Model 3	2.51 (2.31–2.72)	<0.001	1.31 (1.23–1.40)	<0.001

Model 1 = adjusting sex, age, ethnicity and comorbidities (hypertension, coronary heart disease, heart failure, diabetes, chronic kidney disease, chronic pulmonary disease, liver disease, malignant cancer). Model 2 = Model 1 + adjusting blood pressure, laboratory parameters (serum white blood cells, hemoglobin, platelet, sodium and potassium), mechanical ventilation, usage of vasoactive drugs, SOFA score. Model 3 = Model 2 + removing parameters with VIF ≥10 (age, blood pressure, serum hemoglobin, sodium and potassium). CI, confidence interval; OR, odds ratio; SAKI, sepsis-associated acute kidney injury; SOFA, Sequential Organ Failure Assessment.

Using the low-normal category as the reference, hyperphosphatemia was identified as an independent risk factor for developing SAKI, which led to an increased risk by 6.77 to 9.32-fold (OR 7.77–10.32, all *p* < 0.001) in the MIMIC IV and 1.14 to 2.15-fold (OR 2.24–3.15, all *p* < 0.001) in the eICU-CRD cohorts (shown in Table 4). Hypophosphatemia was considered as an independent protective factor for SAKI development in the MIMIC IV cohort (OR 0.68–0.70, all *p* < 0.001), but not statically significance was found in the eICU-CRD cohort (OR 0.85–0.93, all *p* > 0.05).

**Table 4:** Logistic regression analysis for patients in different phosphate categories.

	Unadjusted		Model 1		Model 2		Model 3	
**MIMIC IV database**								
Hypophosphatemia (≤2.7 mg/dL)	0.70 (0.58–0.84)	<0.001	0.68 (0.56–0.82)	<0.001	0.69 (0.57–0.84)	<0.001	0.69 (0.57–0.83)	<0.001
Normal-low (2.7–3.3 mg/dL)	**Reference**							
Normal-high (3.4–4.5 mg/dL)	1.89 (1.69–2.11)	<0.001	2.02 (1.80–2.26)	<0.001	1.97 (1.75–2.22)	<0.001	1.90 (1.69–2.12)	<0.001
Hyperphosphatemia (>4.5 mg/dL)	10.32 (8.25–12.90)	<0.001	9.93 (7.90–12.49)	<0.001	7.77 (6.10–9.90)	<0.001	8.16 (6.44–10.33)	<0.001
**eICU database**								
Hypophosphatemia (≤2.7 mg/dL)	0.93 (0.71–1.21)	0.580	0.89 (0.68–1.17)	0.421	0.85 (0.64–1.13)	0.268	0.88 (0.67–1.16)	0.355
Normal-low (2.7–3.3 mg/dL)	**Reference**							
Normal-high (3.4–4.5 mg/dL)	1.35 (1.03–1.76)	0.026	1.35 (1.03–1.76)	0.029	1.24 (0.93–1.63)	0.138	1.31 (1.00–1.73)	0.050
Hyperphosphatemia (>4.5 mg/dL)	3.15 (2.41–4.12)	<0.001	3.08 (2.34–4.04)	<0.001	2.24 (1.68–3.00)	<0.001	2.47 (1.86–3.27)	<0.001

Model 1 = adjusting sex, age, ethnicity and comorbidities (hypertension, coronary heart disease, heart failure, diabetes, chronic kidney disease, chronic pulmonary disease, liver disease, malignant cancer). Model 2 = Model 1 + adjusting blood pressure, laboratory parameters (serum white blood cells, hemoglobin, platelet, sodium and potassium), mechanical ventilation, usage of vasoactive drugs, SOFA score. Model 3 = Model 2 + removing parameters with VIF ≥10 (age, blood pressure, serum hemoglobin, sodium and potassium). CI, confidence interval; OR, odds ratio; SAKI, sepsis-associated acute kidney injury; SOFA, Sequential Organ Failure Assessment.

Notably, compared to the low-normal category, serum phosphate in high-normal category was independently related to an approximately 95% (OR 1.89–2.02, all *p* < 0.001) increased risk of developing SAKI in the MIMIC IV cohort (shown in Table 4). In the eICU-CRD cohort, serum phosphate in high-normal category also independently associated with a 35% increased risk of SAKI development (Model 1, OR 1.35, 95% CI 1.03–1.76, *p* = 0.026), but the statistical significance disappeared after adjusting for potential confounders (Models 2–3, *p* ≥ 0.05).

### 3.5 Subgroup analysis

**Figure 3:**
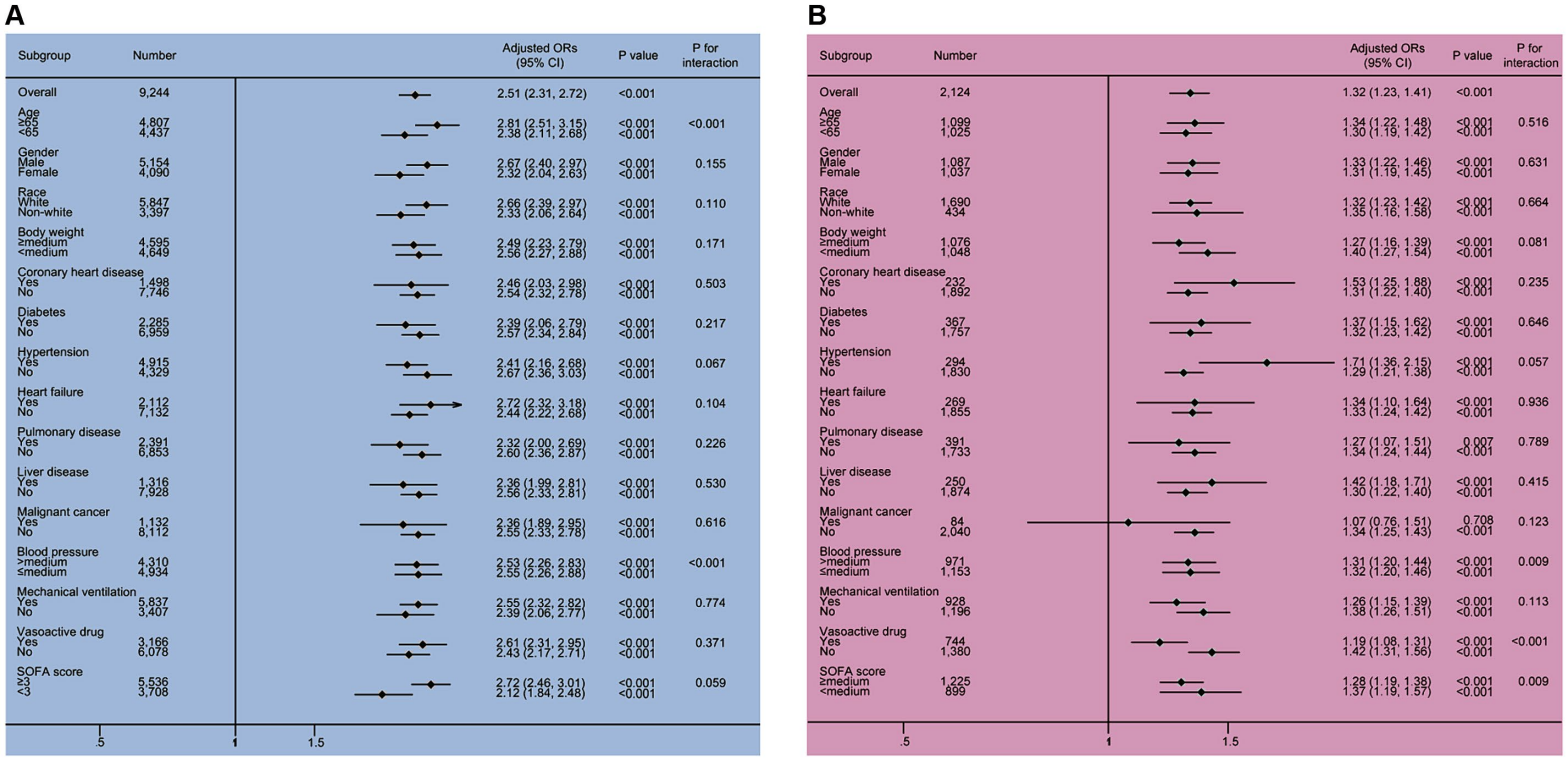
The results of the subgroup analysis in the MIMIC IV and eICU-CRD cohorts. Two significant interactions, including phosphate × age and phosphate × MBP, were found in the MIMIC IV cohort, and three interactions, including phosphate × MBP, phosphate × vasoactive drug and phosphate × SOFA score were identified in the eICU-CRD cohort. All subgroup results supported that elevated serum phosphate was associated with the increased risk of SAKI occurrence (all OR >1). CI, confidence interval; OR, odds ratio; SOFA, sequential organ failure assessment.

In the subgroup analysis (as shown in Figure 3), serum phosphate levels were independently were found to be independently associated with the risk factor of SAKI development in all subgroups of the MIMIC IV and eICU-CRD cohorts (OR >1, *p* < 0.001), except for the malignant cancer (+) subgroup from the eICU-CRD cohort (OR 1.07, 95% CI 0.76–1.51, *p* = 0.708).

In the MIMIC IV cohort, two interactive factors between serum phosphate and the risk for developing SAKI were identified, phosphate × age and phosphate × MBP (all *p* for interaction <0.001). In the eICU-CRD cohort, significant interactions were observed between phosphate and MBP (*p* for interaction = 0.009), phosphate and vasoactive drug (*p* for interaction = 0.001), as well as phosphate and SOFA (*p* for interaction = 0.009).

### 3.6 Assessment of clinical predictive value of serum phosphate levels using ROC curve

**Figure 4:**
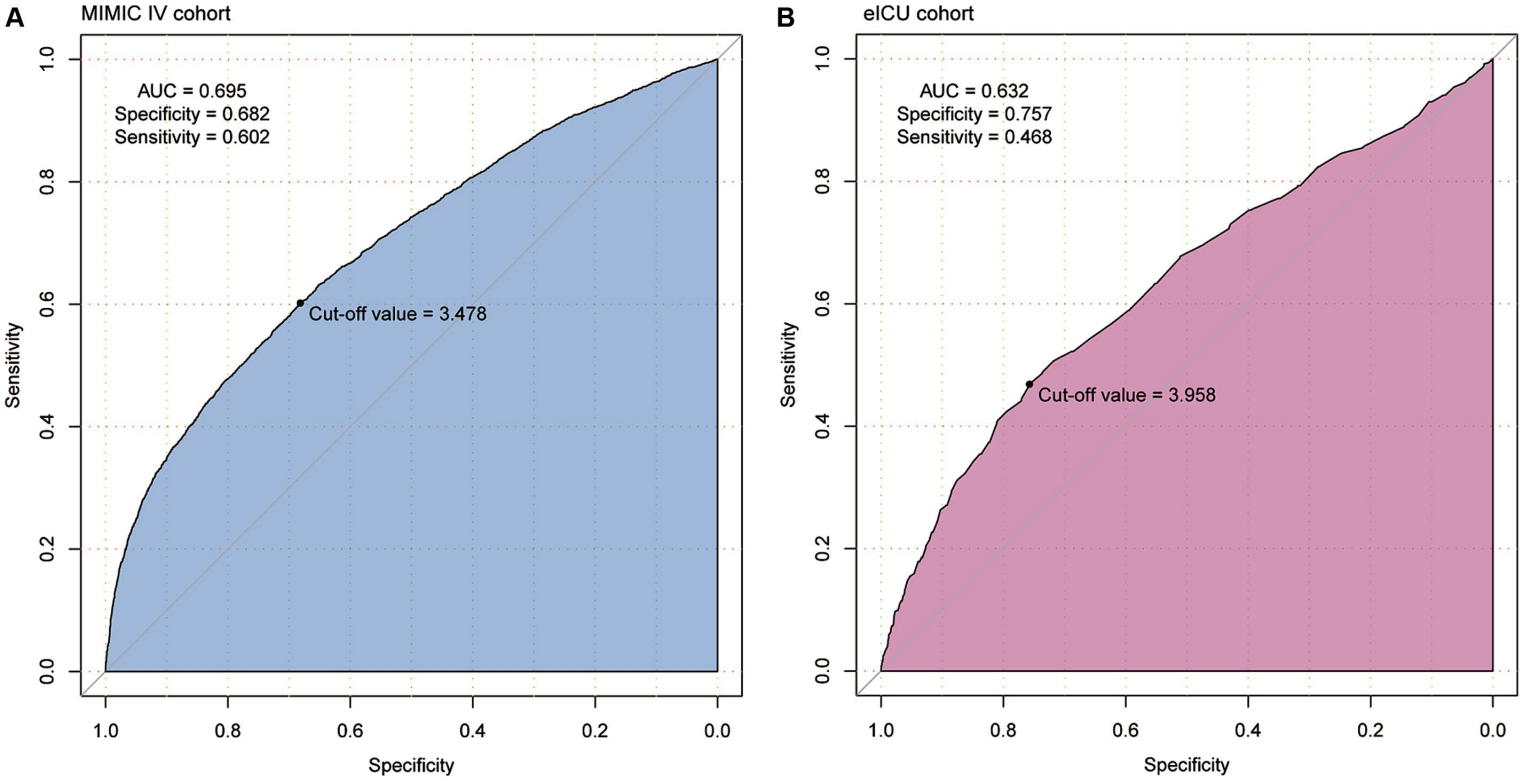
The clinical predictive value of serum phosphate for the development of SAKI was identified in patients from MIMIC IV **(A)** and eICU-CRD **(B)** cohorts using the receiver operating characteristic (ROC) curves. Serum phosphate showed moderate clinical value for predicting the development of SAKI (AUC = 0.695 and 0.632).

ROC analysis revealed a moderate clinical predictive value of serum phosphate levels for SAKI incidence, with an AUC of 0.695 and 0.632 in the MIMIC IV and eICU-CRD cohorts, respectively. Further details can be found in Figure 4.

## 4 Discussion

In this multicenter study, we were able to fully validate the association between serum phosphate and the development of SAKI in patients with sepsis using two independent databases. Our findings demonstrated that elevated serum phosphate levels were significantly associated with an increased risk of developing SAKI. Not only hyperphosphatemia (>4.5 mg/dL), but also high-normal phosphate levels within the normal reference range, were identified as independent risk factors for SAKI. Furthermore, hyperphosphatemia was associated with an increased risk of both hospital and ICU mortality. Although hypophosphatemia (<2.7 mg/dL) appeared to be a protective factor based on our results, this relationship did not hold consistently across different cohorts. Nonetheless, it is worth noting that even within the normal reference range, high-normal phosphate levels were found to be a significant risk factor for SAKI, which should be a cause for concern in clinical practice. To the best of our knowledge, few studies have systematically investigated the potential relationship between serum phosphate and the risk of developing SAKI. Thus, our data provide valuable evidence supporting the use of serum phosphate as a biomarker for the early diagnosis of SAKI.

The clinical value of serum phosphate has increasingly drawn attention. While several studies have investigated the potential association between abnormal phosphate levels and the prognosis of sepsis, most of them have focused on mortality rather than AKI. In fact, the relationship between serum phosphate and the prognosis of sepsis so far remains controversial. For instance, Black et al. (15) demonstrated that the highest quartile of phosphate (>4.0 mg/dL) was associated with increased odds of mortality. Similarly, Haider et al. (16) reported that hyperphosphatemia was an independent risk factor for mortality in critically ill patients, increasing the risk of death by 229% (OR 3.29, *p* < 0.001). The association between hyperphosphatemia and increased all-cause mortality has also been observed in cohorts of COVID-19 (17), severe burns (18) and pancreatitis cohorts (19). However, these associations have been negated in cohorts of patients undergoing coronary artery bypass grafting and hemodialysis (20, 21). Approximately 20% of critically ill patients experienced hypophosphatemia (22), and this incidence rates were 14.0 and 29.0% in the present study. Hypophosphatemia, on the other hand, has been found to be a general biomarker of severity in critically ill patients and may increase the risk of 28 days mortality by 50% (OR = 1.5) (23). Severe hypophosphatemia (<1 mg/dL) in sepsis patients has been shown to increase the risk of death by 11.2-fold (11). However, there are conflicting reports regarding the association between hypophosphatemia and mortality rates in sepsis patients, with some studies suggesting a decreased risk of mortality (9, 10, 24). These discrepancies in findings can be attributed to differences in target populations, timing of phosphate measurement, and the threshold values used to define abnormal phosphate levels. A recent meta-analysis including 38,320 patients with sepsis or septic shock from 10 researches confirmed a significant association between increased serum phosphate levels and increased risk of mortality (RR = 1.46, 95% CI 1.22–1.74, *p* < 0.001). However, no significant association was found between low serum phosphate levels and mortality risk (*p* = 0.588) (25). It is important to note that pooled results from meta-analyses should be interpreted with caution due to the inherent heterogeneity of the original data.

As another important endpoint in sepsis, few studies have investigated the clinical value of serum phosphate in predicting AKI in the sepsis cohort. A single-center retrospective study including 5,036 participants showed that the admission serum phosphate levels ≥4.4 mg/dL were associated with an increased risk of developing AKI (OR 1.72, 95% CI 1.20–2.47) compared to a reference phosphate level of 2.4–2.9 mg/dL. However, serum phosphate levels <4.4 mg/dL showed no association with the risk of developing AKI (26). Another study by Moon et al. (27) demonstrated that the third and fourth quartiles of serum phosphate levels were associated with a 40% (OR = 1.4) and 180% (OR = 2.8) increased risk of developing AKI, respectively, compared to the first quartile. This was observed in hospitalized patients both with and without chronic kidney disease (CKD). Furthermore, increased serum phosphate levels have been identified as predictive biomarkers for the development of AKI in conditions such as tumor lysis syndrome (28, 29) and rhabdomyolysis (30). In present study, we confirmed the predictive value of hyperphosphatemia for an increased risk of developing AKI in sepsis patients, which is partly consistent with previous studies. We observed a nearly positive relationship between serum phosphate levels and the risk of SAKI development. Hyperphosphatemia was consistently identified as a significant risk factor for SAKI development in two independent cohorts. Hypophosphatemia, on the other hand, was negatively associated with the risk of SAKI development, but statistical significance was only found in the MIMIC IV cohort. The lack of significance in the eICU-CRD cohort may be attributed to the smaller sample size. Interestingly, our findings also revealed that serum phosphate levels within the normal reference range could have opposite predictive values for developing AKI in septic patients. Similar observations have been reported in previous studies indicating that even slight increases in serum phosphate levels within the normal range are associated with an increased risk of vascular calcification, arterial stiffness, and microvascular dysfunction (31, 32). Thus, our findings suggested that except for hyperphosphatemia, phosphate levels even within the normal range also requires much attention in septic patients.

The exact mechanism by which phosphate affects the occurrence of SAKI is still unclear, and it is challenging to elucidate the interrelationship between serum phosphate and kidney injury in septic cohort. Ikeda et al. (33) reported that in the LPS-induced septic mouse model, the protein expression level of type II sodium-dependent phosphate cotransporter (Npt2a) in the brush border membrane was significantly decreased rapidly after LPS injection. The decrease in Npt2a in the brush border membrane can lead to abnormal renal proximal tubular reabsorption of phosphate, which is thought to be the primary mechanism of phosphate regulation (34). They also found that some hormones associated with phosphate regulation, such as intact parathyroid hormone (PTH) and intact fibroblast growth factor 23 (FGF23), were also upregulated in the septic status (33). On one hand, the toxic effects of excessive phosphate overload would contribute to organ damage, including kidney injury. Phosphate has been shown to mediate inflammatory reactions, oxidative stress responses, cytotoxic effects and chronic vascular calcification (35). Phosphate is an independent risk factor for the presence of an inflammatory status, and its levels are positively correlated with classic inflammatory biomarkers such as C-reactive protein and interleukin-6 (36). Dietary phosphate overload is associated with the infiltration of pro-inflammatory M1 macrophages and elevated levels of TNF-α (37, 38). In addition, oxidative stress damage has been reported as a critical method of phosphate-mediated organ injury (39, 40). Phosphate can also aggravate endothelial cell apoptosis by regulating the cleavage of caspase-3 protein (38). Abnormally high extracellular phosphate levels cause cell proliferation, epithelial-mesenchymal transition, endoplasmic reticulum stress and apoptosis through the regulation of MAPK and AKT signaling pathways (41). Notably, the imbalance of inflammatory response, excessive oxidative stress response, endothelial and epithelial injury induced by infection also has been shown to be involved in the occurrence of sepsis-associated organ dysfunction and damage (42). Kidney tissue would be more susceptible to damage from the combined effect of sepsis and phosphate toxicity. On the other hand, as an essential regulator of serum phosphate levels, damage to renal tissue may lead to abnormal excretion and reabsorption of phosphate. In addition, during cell death and impaired membrane integrity, intracellular phosphate is released into the systemic circulation, resulting in elevated serum phosphate levels. This potential mechanism may explain the lower risk of developing AKI in patients with hypophosphatemia, suggesting less tissue destruction in the kidneys. As a retrospective observational study, we are not able to explore the potential mechanisms or diseases pathogeneses underlying the associations we found. Further studies are needed to explore these potential mechanisms and gain a better understanding of how phosphate contributes to kidney injury, especially in the acute phase of septic patients.

It is an interesting future question whether modulation of phosphate levels will improve the outcomes of SAKI, although limited research has investigated this issue. Dietary phosphate overload is known to be detrimental, as it may accelerate the inflammatory response and the formation of calcium phosphate crystals leading to vascular disease and organ dysfunction (37, 38, 43). Recently, Hamid et al. (44) found that a phosphate-restricted diet had a positive effect on improving survival rates in mice with AKI by effectively downregulating the expression levels of FGF23, PTH and calcitriol. The phosphate restriction diet also prevented metabolic acidosis, hypocalcemia, hyperkaliemia and cardiac electrical abnormalities. This is a novel approach to AKI treatment, but its therapeutic effect in the SAKI populations should be further demonstrated. In addition to the regulation of circulating phosphate levels, the maintenance of local intestinal phosphate levels has also been reported to be associated with the risk of developing sepsis. Maintaining intestinal phosphate abundance and inhibiting local phosphate depletion could help to inhibit the development of entheogenic sepsis (45, 46). This may be a useful alternative in the treatment of sepsis and its complications.

Our study has several strengths. Firstly, we were able to systematically investigate the association between serum phosphate and the development of AKI in sepsis cohorts, which had not been done previously. Secondly, we took various confounding factors into account, including CKD, dialysis, and parathyroid dysfunction. Since these conditions can significantly influence serum phosphate levels, we excluded patients with CKD, hyperparathyroidism, hypoparathyroidism, and those who received dialysis therapy prior to the diagnosis of sepsis. Additionally, we adjusted for other potential confounders such as blood pressure, vasoactive drugs, and comorbidities in our logistic models. Thirdly, by using phosphate results charted before the diagnosis of sepsis, rather than within the first 24 or 48 h after diagnosis, we aimed to minimize any unexpected effects of AKI on serum phosphate levels. Lastly, we utilized data from two independent databases, MIMIC and eICU-CRD, to enhance the reliability and generalizability of our findings.

Despite these strengths, there are some limitations that should be acknowledged, and the results should be interpreted with caution. Above all, although we made efforts to minimize the impact of confounding factors, retrospective studies are inherently susceptible to unknown confounders that may influence our conclusions. Additionally, the use of older databases may introduce bias due to the evolving sepsis guidelines and management practices over time. What’s more, the sample size of our study may still be insufficient, and there is a possibility of small sample bias and selection bias. Since we focused on hospitalized patients, Berkson bias cannot be avoided. In addition, as serum phosphate is a dynamic biomarker, using a single value may not fully capture the relationship over time. Finally, although we emphasize the predictive value of blood phosphate on the development of SKAI, it is still unclear whether it has clinical value in distinguishing SAKI from general AKI. Further high-quality clinical trials and trajectory analyses are needed to evaluate the effect of serum phosphate levels on the incidence of AKI in sepsis patients. Research into the differential predictive value of blood phosphate in SAKI and other types of AKI is also crucial.

## 5 Conclusion

Our study provides evidence supporting the significant association between the elevated serum phosphate levels and the increased risk of developing SAKI in septic patients. Additionally, attention should be given to higher phosphate levels within the normal range, as they may still contribute to the risk of AKI development in septic patients. The protective role of hypophosphatemia remains undetermined. Monitoring serum phosphate levels may offer valuable insights for early detection and management of SAKI in septic patients.

## Data availability statement

Publicly available datasets were analyzed in this study. This data can be found here: the data used in this study were obtained from the publicly available MIMIC IV (https://physionet.org/content/mimiciv/2.2/) and eICU-CRD (https://physionet.org/content/eicu-crd/2.0/). All details of our raw data and codes we used can be obtained from the corresponding author on reasonable request.

## Ethics statement

This present study utilized data from the MIMIC-IV and eICU-CRD databases. The Institutional Review Boards of the Massachusetts Institute of Technology (Cambridge, MA, United States) and the Beth Israel Deaconess Medical Center (Boston, MA, United States) provided ethics approval for the use of the MIMIC-IV database. Additionally, using eICU-CRD database for secondary analysis can be exempt from institutional review board approval (available at: https://eicu-crd.mit.edu/about/acknowledgments/). The studies were conducted in accordance with the local legislation and institutional requirements. Written informed consent for participation was not required from the participants or the participants’ legal guardians/next of kin in accordance with the national legislation and institutional requirements.

## Author contributions

YF: Conceptualization, Data curation, Formal analysis, Methodology, Project administration, Software, Validation, Visualization, Writing – original draft. YZ: Data curation, Formal analysis, Software, Validation, Visualization, Writing – original draft. XZ: Conceptualization, Funding acquisition, Project administration, Supervision, Writing – review & editing.

## Figures and Tables

**Table:** 

AKI	Acute kidney injury
ATP	Adenosine 5′-triphosphate
AUC	Area under the ROC curve
BIDMC	Beth Israel Deaconess Medical Centre
BUN	Blood urea nitrogen
CKD	Chronic kidney disease
CRRT	Continuous renal replacement therapy
eICU-CRD	eICU Collaborative Research Database
ICUs	Intensive care units
LOS	Length of stay
MIMIC IV	Medical Information Mart for Intensive Care IV
NIH	National Institutes of Health
RCS	Restricted cubic spline
ROC	Receiver operating characteristic
SAKI	Sepsis associated acute kidney injury
SCr	Serum creatinine
SD	Standard deviation
STROBE	Strengthening the Reporting of Observational Studies in Epidemiology
WBC	White blood cells
